# BCG Against SARS-CoV-2: Second Youth of an Old Age Vaccine?

**DOI:** 10.3389/fphar.2020.01050

**Published:** 2020-07-09

**Authors:** Siya Kamat, Madhuree Kumari

**Affiliations:** Department of Biochemistry, Indian Institute of Science, Bangalore, India

**Keywords:** COVID-19, BCG vaccine, trained immunity, clinical trials, IL-1β

## Abstract

The sudden outbreak of the COVID-19 pandemic, caused by SARS-CoV-2, has put the whole world into a difficult situation, asking for the immediate development of therapeutics and vaccines against the disease. Bacillus Calmette–Guérin (BCG), an attenuated strain of *Mycobacterium bovis*, has been administered for decades in many countries against tuberculosis. Today, when a solution against SARS-CoV-2 is urgently needed, the BCG vaccine has again come into the limelight owing to its earlier prevention of non-specific diseases. Data suggest a higher mortality rate of COVID-19 in non-BCG vaccinated countries, whereas the nations opting for BCG immunization have a comparatively lower mortality rate. The BCG vaccine is known to induce ‘trained immunity’ and generate ‘non-specific’ heterologous immune responses. It can confer anti-viral immunity by eliciting the production of pro-inflammatory cytokines, IL-6, TNF-α, IFN-γ, and IL-1β. Though the initial results look promising, a long trail still needs to be followed to avoid false promises. The accuracy of nationwide data, the role of an already activated immune system against ‘cytokine storms’, optimization and timing of vaccine dosage, and balancing demand-supply are some of the relevant issues that must be resolved before reaching a final conclusion.

## SARS-CoV-2: A Modern Tragedy

The pandemic that emerged in China, caused by severe acute respiratory syndrome coronavirus-2 (SARS CoV-2), is turning into the most devastating global event of the 21^st^ century (Gorbalenya et al., 2020). The disease COVID-19 is characterized by symptoms of fever, cough, shortness of breath, and muscle ache. Severe cases exhibit pneumonia-like symptoms in the lower respiratory tract with a bilateral diffuse alveolar damage (DAD), pulmonary edema and hyaline membrane formation, indicative of acute respiratory distress syndrome (ARDS), and characteristic syncytial cells in the alveolar lumen which hijack other vital organs (Zhu et al., 2020). The virus makes its hallmark entry using the human angiotensin-converting enzyme 2 (ACE2) receptor by employing its glycosylated spike (S) protein (Hoffmann et al., 2020). The cryo-EM structure analysis by Wrapp et al. (2020) described the binding affinity of the S protein to ACE2 as 20 times higher in SARS-CoV-2 than its counterparts, which contributes to its high contagiousness.

Currently, broad-spectrum antibiotics and anti-viral drugs like interferon α (IFN-α) and lopinavir/ritonavir are utilized (Chakraborty et al., 2020; Dong et al., 2020). Patients who have been given convalescent plasma therapy to treat COVID-19 have shown an appreciable recovery rate (Chen et al., 2020). Nevertheless, to efficiently combat this pandemic, a vaccine is the most promising weapon (Bhattacharya et al., 2020; Peeples, 2020). A lot of scientific interest is stirring over the effectiveness of the bacillus Calmette-Guérin (BCG) vaccine against SARS- CoV-2. In 1993, WHO declared tuberculosis (TB) a global health emergency. An estimated 10 million people get infected and 1.2 million die every year. Hence, the BCG vaccine is prescribed against TB (Tameris et al., 2019).

## How Does the BCG Vaccine Work?

After 84 years of BCG use in clinical settings, it was established as a blockbuster therapy to overturn highly infective *Mycobacterium tuberculosis* (*Mtb*). In the current pandemic, it is hypothesized that the BCG vaccine can shield against SARS-CoV-2. Thus, it is imperative to understand the underlying immune mechanisms of BCG against *Mtb* (Tameris et al., 2019). Verrall et al. (2020) mention that the protective effect of BCG was dose-dependent and not absolute, i.e. protection was at a maximum against minimum levels of pathogen exposure. Secondly, the authors also mentioned that the effectiveness of the vaccine wanes with age, with no protection in subjects >38 years of age. Upon first contact with *Mtb*, the innate immune system is responsible for the “early clearance”. However, the BCG vaccination facilitated the elimination of the pathogen through “trained immunity” (Arts et al., 2016). This is an effective non-specific immune response generated against a second infection, independent of the initial antigen (Cirovic et al., 2020). To decipher the basis of trained immunity, various studies on mice and humans have revealed that the vaccination brings about an epigenetic reprogramming in monocyte-derived macrophages (MDM), which stimulates the production of cytokines like IFN-γ, TNF-*α*, IL-1β, IL-10, and IL-12. Out of these, IL-1β represents the endogenous mediator which brings about the long-term effective reprogramming in bone marrow progenitors. This, in turn, affects the survival of intracellular *Mtb*, thereby preventing a systemic infection (Arts et al., 2018a). This pleiotropic response of BCG brings about a 30% decrease in infant mortality (Shann, 2010). Due to these features, BCG vaccination can also protect against a non-related viral infection and certain carcinomas (Arts et al., 2016).

## COVID-19 and TB: Which is More Deadly?

The invaders, *Mtb* and SARS-CoV-2, transmitted *via* droplet nuclei of aerosols generated by infected beings, impact the lungs and eventually compromise the host immunity. *Mtb* has a longer incubation time of 3-9 months or 1-2 years, while SARS-CoV-2’s incubation time is a few days. However, both of these diseases display similar symptoms (Dara et al., 2020). Alveolar macrophages are presumed to first encounter *Mtb*, although neutrophils, monocyte-derived macrophages, and dendritic cells are also observed to ingest the pathogen and hence play a significant role in the infection. The phagocytic cell receptors involved in the interaction are C-type lectin receptors, scavenger receptors, and complement receptors that bind to mycobacterial cell wall lipoglycans and mannose-capped lipoarabinomannans. This determinant step of the infection influences the phagosome maturation and cytokine signaling (Philips and Ernst, 2012). Uptake *via* specific receptors determines the intracellular fate of the pathogen, i.e. whether it will resist phagosome maturation or result in delivery into the lysosome. *In vivo*, several receptors are co-expressed on the same cells, because of which *Mtb* uptake occurs through the cooperation of relevant receptors. Hence, unlike other pathogens that develop strategies to dodge entry into host phagocytic cells, *Mtb* uses multiple routes to enter and exploit host phagocytic cells (Kang et al., 2005). The long-term victory of the pathogen is also contributed to by interfering with autophagy and granuloma formation (Volkman et al., 2010). The host innate immune system defends against the infection, followed by the induction of CD4 and CD8 T cell responses, which eventually proves unsuccessful in combating *Mtb* (Chen et al., 2007).

SARS-CoV-2, Middle East Respiratory Syndrome (MERS)-CoV, and SARS-CoV display differing statistics. The mortality rate of MERS-CoV was estimated at 34%, whilst that of SARS-CoV was 9% (Henrickson, 2020). This could be attributed to ACE2, which is the specific receptor utilized by SARS-CoV-2. Such cells are found in the lungs, GI and renal tract, and the heart, which are the major arenas for organ failure (Park et al., 2020). The immune response generated by this infection is observed to be two-phased. In the early stage, an adaptive immune response is employed to eradicate the virus and inhibit further progression. Hence, specific anti-viral immunity is elicited if the host has a better human leukocyte antigen (HLA) background, maintains good health, and boosts their immunity. Thus, differential susceptibility is seen in various individuals. But, when this first phase immune response is impaired, the virus is observed to initiate massive destruction in tissues expressing its receptor, ACE2. This damage results in the induction of lung inflammation, mediated by the cytokine storm initiated by macrophages and granulocytes. Hence, deaths that are reported in severe stages are mainly due to lung inflammation. It has also been observed that defective production and regulation of hyaluron results in fluid accumulation in the lungs. This is also associated with the secretion of IL-1 and TNF, which are known to be strong inducers of HA-synthase 2 in lung alveolar and epithelial cells (Prompetchara et al., 2020).

## The Response of BCG-Vaccinated Individuals to SARS-CoV-2?

While most experts converge on the efficacious properties of BCG against TB, its role in SARS-CoV-2 has resulted in a lot of debate and speculation. Various studies have reported on the non-specific effects of BCG against the respiratory syncytial virus, yellow fever, herpes simplex virus, and human papillomavirus, which in some way supports the claim that this vaccine may indeed have some capacity to fight severeCOVID-19 infection (Hegarty et al., 2020).

Randomized clinical trials in Columbia (NCT04362124), the Netherlands (NCT04328441), Cape Town, South Africa (NCT04379336), Egypt (NCT04350931), Australia (NCT04327206), the USA (NCT04348370), Denmark (NCT04373291), and France (NCT04384549) have been listed on US-NLM to evaluate the performance of the BCG vaccine in the protection of healthcare workers who come in to direct contact with COVID-19 patients. They also intend to check how the vaccine can activate the immune system against SARS-CoV-2, which could prevent severity and hence reduce fatality. The trials were initiated based on the observation that Columbia’s first case was reported on March 6^th^, 2020, even though the virus was already circulating much earlier. It is also known that Latin America and other countries like Italy and Spain differ significantly in BCG vaccination rates.

Another randomized trial in the Netherlands (NCT04417335) is exploring the preventive effects of BCG vaccination in the elderly. Other ongoing trials include NCT04347876 (Egypt) and NCT04369794 (Brazil), which are evaluating the impact of previous or current BCG exposure on COVID-19 and the elimination of SARS-CoV-2 at different time points.

In 2017, the Hellenic Institute for the Study of Sepsis, Greece, initiated the ACTIVATE (A Randomized Clinical trial for enhanced Trained Immune responses through Bacillus Calmette-Guérin VAccination to prevenT infections of the Elderly) trial (NCT03296423), which is currently in phase 4. In this trial, 200 hospitalized elderly patients were administered with single doses of a BCG vaccine or a placebo on the day of discharge and monitored for 12 months; the last patient is scheduled for August 2020. The interim analysis of this study revealed a 53% decrease of the appearance of new infections and an 80% decrease of common respiratory tract infections in the BCG group compared with the placebo group. These results formed the rationale for the next trial (NCT04384549) of the same group, which is currently in phase 3, evaluating the efficacy of BCG vaccinations as a prophylactic tool against COVID-19 in healthcare workers.

An inverse correlation between BCG vaccination coverage and COVID-19 associated morbidity and mortality has been reported from Japan too, despite its population density being 2.4 times higher than New York City (Iwasaki and Grubaugh, 2020). Japan, Korea, India, and Russia have mandatory childhood BCG vaccination programs with low per capita deaths as compared to the USA, Netherlands, and France (Redelman-Sidi, 2020). This information comes from the BCG World Atlas which states that nations like China, India, Japan, and other Asian countries follow universal BCG vaccination programs while Spain, France, and Switzerland have discontinued this program due to the low risk of developing TB (Zwerling et al., 2011). Similarly, due to low-risk populations, the USA, Italy, and the Netherlands, have not adopted the vaccination program. The results of these trials will help to understand if indeed there is any BCG-induced resistance in combating severe cases of SARS-CoV-2 (Hegarty et al., 2020). Bayler College of Medicine in the US is also assessing BCG activity on COVID-19 patients (https://www.bcm.edu/news/infectious-diseases/testing-tuberculosis-vaccine-for-covid-19). MD Anderson and National Cancer Institute, USA, are currently in phase III of randomized clinical trials to test Tokyo-172 strain BCG in treating bladder cancer patients (NCT03091660). The results so far have produced interesting observations that indicate stimulation of the immune system when BCG is instilled into the bladder. They are trying various versions of BCG with vaccine therapy, which could potentially prevent bladder cancer relapse (https://clinicaltrials.gov/ct2/show/NCT03091660). Another study conducted in perinatal HIV-exposed African infants (NCT02062580) reported that BCG vaccination induces an increase in activated HIV target T cells CCR5^+^CD4^+^. Hence, the authors suggested an optimal vaccine timing to reduce the dangers of HIV transmission while maintaining the efficacy of BCG against tuberculosis (Gasper et al., 2017).

The pediatric populations of China and other Asian countries demonstrated a significant recovery rate and mild symptoms from SARS-CoV-2. This is being attributed to regular immunizations with BCG which generated a trained immunity (Cao et al., 2020).

Israel had a 90% BCG coverage between 1955-1982; since 1982 the vaccine has been administered only to immigrants from countries with a high prevalence of TB. In a cohort of Israeli subjects aged 35-41 years, no significant difference was observed in the protective effect of BCG vaccines among the vaccinated COVID-19 positive (11.7%) vs the unvaccinated COVID-19 positive (10.4%) individuals, with no deaths in both the groups. The results of this study do not support the protective effect of BCG vaccination in childhood against COVID-19 (Hamiel et al., 2020).

However, to demonstrate the ecology of this vaccine, confounding variabilities in the detection of cases, the temporal nature of the viral spread, the impact of dynamic populations, age, demographics, variable death certification and reporting times, and some differences between the planned and actual BCG execution within countries need to be exhaustively analyzed and explored to quantify whether BCG is influential in the current pandemic (Hegarty et al., 2020).

## The Correlation of Mechanisms Between the BCG Vaccine and SARS-CoV-2

**Figure 1:**
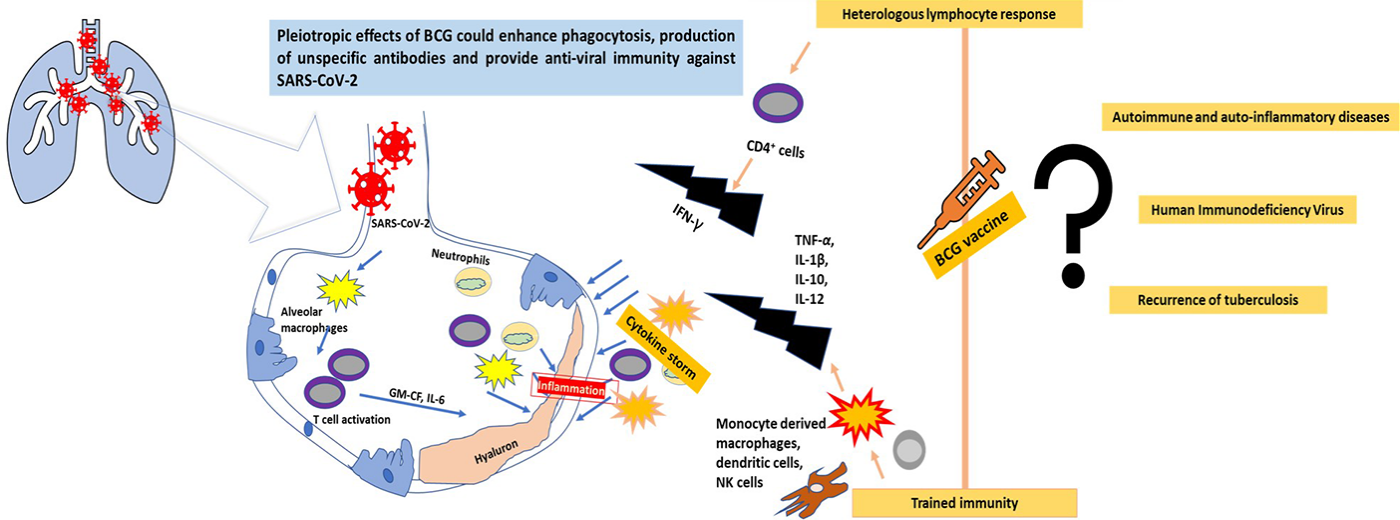
The progression of SARS-CoV-2 infection in the lungs and the possible role of the BCG vaccine in combating the virus through trained immunity and a heterologous immune response.

Though the BCG vaccine is given against a mycobacterium, it is known to generate ‘heterologous effects’ and ‘trained immunity’ against a myriad of viral pathogens (Curtis et al., 2020). Epidemiological data suggest that BCG vaccination in infants can significantly reduce the mortality rate, due to protection against heterologous infections caused by increasing the population of antigen-independent T and B cells (Silvia et al., 2018; Redelman-Sidi, 2020). Trained immunity can be seen as a non-specific increase in the innate immune responses that alters the metabolic and epigenetic configuration of the immune system (Netea et al., 2020). Trained immunity has gained a lot of importance over the last two decades owing to its immune-modulatory actions against cancer and viral, fungal, and bacterial diseases (Redelman-Sidi, 2020). Histone modification, chromatin remodelling, acetylation and methylation reprogramming, and changes in cholesterol and amino acid metabolism are responsible for the development of trained immunity in monocytes, macrophages, dendritic cell, T cells, and NK cells, resulting in enhanced production of pro-inflammatory cytokines, IL-6, TNF-α, and IFN-γ (Uthayakumar et al., 2018; Covián et al., 2020). Many of the induced cytokines, interferons, interleukin-1, and TNFs are known to play a vital role in the suppression of viral pathogens (**Figure 1**). Increased production of IL-1β after a BCG vaccination can provide anti-viral immunity (Covián et al., 2020). Moorlag et al. (2018) and Arts et al. (2018a) have demonstrated the role of induced non-specific IL-1β associated with trained immunity in decreasing the yellow fever viremia. Kleinnijenhuis et al. (2014) found that heterologous production of Th1 (IFN-γ) and Th17 (IL-17 and IL-22) immune responses were potentially elevated against non-mycobacterium pathogens even one year after BCG vaccination. A peritoneal macrophage dependent, IFN-γ independent protection against vaccinia virus and herpes simplex virus type 2 has earlier been reported after immunization with a BCG vaccine (Starr et al., 1976).

Currently, no established mechanism of BCG can be reported for protection against SARS-CoV-2. However, the earlier successful role of BCG vaccines against multiple viral diseases and its reduction in their overall mortality means certain mechanisms employed by BCG vaccines can be presumed to have probable anti-SARS-CoV-2 actions. BCG vaccines enhance phagocytosis of airborne pathogens and increases the population of the memory T cells in the lungs (Perdomo et al., 2016; Covián et al., 2019). Leentjens et al. (2015) in their study observed an increase in unspecific functional antibodies titer and enhanced seroconversion against Influenza -A(H1N1) virus after injection of a BCG vaccine. As COVID-19, H1N1, and tuberculosis are all diseases associated with pulmonary infections, these strategies of BCG have a high probability to work against SARS-Co-2 as well. Stensballe et al. (2005) in their randomized trials in Guinea-Bissau found that BCG-administered girls were 3 times more immune to acute lower respiratory tract infections and respiratory syncytial viruses than their non-BCG administered counterparts. The BCG vaccine has been shown to induce heterologous lymphocyte responses against non-specific antigens by increasing CD4^+^ population of cells and is associated with an increase of CD4^+^ and CD8^+^ memory cells (Moorlag et al., 2019), which can help to combat COVID-19. It has also been shown to modulate adaptive immune responses and increase production of IgG and long-lived memory B cells. Though no direct targets could be linked between BCG vaccines and SARS-CoV-2, the similar site of infection and enhanced innate and acquired immunity attributing to trained immunity means the vaccine holds potential in this grim battle against COVID-19. However, the conversion of this ‘ray of hope’ into ‘factual figures’ is incomplete without investigating all areas of the BCG vaccine in detail.

## Paths to be Followed

Though the initial results and country-wide data of BCG vaccinations looks promising, a long path needs to be followed (**Figure 1**). Due to the current scenario in which the rate of testing of COVID-19 in developed and developing countries differs remarkably, linking this data with BCG vaccinations can provide false results. Moreover, country-wide data cannot reflect individual data or randomized clinical trials. The WHO also does not recommend BCG vaccination for COVID-19 prevention (WHO Bulletin, 2020).

‘Cytokine storm’ is a major mechanism responsible for the respiratory failure with SARS-CoV-2 (Prompetchara et al., 2020). Today, where a therapeutic drug is urgently needed to block cytokine production and TNF α-signalling to prevent COVID-19, results of trained immunity induced by BCG vaccinations remains uncertain. Arts et al. (2018b) have hypothesized on the adverse effects of trained immunity after a BCG vaccination against autoimmune diseases and auto-inflammatory disorders due to an already activated innate immune system. Jensen et al. (2017) concluded that trained immunity can be a double-edged sword against Human Immunodeficiency Virus (HIV). Activation of CD4^+^ T, CD8^+^ T, and B cells after BCG vaccination can aggravate the symptoms of HIV and can pose a potential threat in the areas where tuberculosis and HIV need to be simultaneously eradicated. Thobakgale et al. (2017) observed that after antiretroviral therapy (ART) and BCG vaccination, recurrence of tuberculosis increased due to an increased production of IL-1β. Without randomised clinical trials and proper results, it is too early to generate false promises related to the BCG vaccine.

Most of the studies have advocated for the role of the BCG vaccine in reducing mortality at an early age (Silvia et al., 2018). The effectiveness of this vaccine after decades of its administration remains doubtful without scientific interpretation. Even if the BCG vaccine is effective against SARS-CoV-2, the type of vaccine, the formulations using recombination methods, the potential to induce non-specific trained and heterologous immunity, and the mode and time of administration need to be evaluated.

BCG is a vaccine given to infants under immunization programs, especially in developing countries, and is one of the most common immunotherapies provided to treat early-stage bladder cancer (Redelman-Sidi, 2020). A sudden surge in the demand for BCG vaccines after its hype may create an imbalance in supply and demand, especially when the suppliers are limited. The COVID-19 pandemic is a critical situation, wherein small pieces of information can generate great levels of interest, so a careful evaluation of the efficacy of the BCG vaccine against SARS-CoV-2 needs to be evaluated before creating hope.

## Conclusions

The BCG vaccine given against tuberculosis has acquired new interest because of its probable anti-SARS-CoV-2 action. Some statistical data depict a higher death rate of COVID-19 in non-BCG vaccinated countries than in BCG-vaccinated nations. Though the results of clinical trials are still awaited, this has created much hype. BCG vaccines provide trained and heterologous immunity against multiple diseases by eliciting non-specific innate and adaptive immunities. Tuberculosis and COVID-19, both pulmonary diseases, have again supported the role of trained immunity induced by the BCG vaccine against SARS-CoV-2. However, it is too early to conclude the results without randomized clinical trials and further scientific research.

## Author Contributions

SK and MK collected the literature, formulated the draft, and provided intellectual input for the manuscript. SK and MK critically reviewed the manuscript draft.

## Conflict of Interest

The authors declare that the research was conducted in the absence of any commercial or financial relationships that could be construed as a potential conflict of interest.
